# P-1207. Resurgence of Respiratory Viral Infections Among Children Hospitalized in a Tertiary Care Hospital in Thailand After the COVID-19 Pandemic

**DOI:** 10.1093/ofid/ofae631.1389

**Published:** 2025-01-29

**Authors:** Tavitiya Sudjaritruk, Oramai Mueangmo, Jutamad Saheng, Tanachot Chaito, Suphara Manowong

**Affiliations:** Faculty of Medicine, Chiang Mai University, Chiang Mai, Chiang Mai, Thailand; Faculty of Medicine, Chiang Mai University, Chiang Mai, Chiang Mai, Thailand; Faculty of Medicine, Chiang Mai University, Chiang Mai, Chiang Mai, Thailand; Faculty of Medicine, Chiang Mai University, Chiang Mai, Chiang Mai, Thailand; Faculty of Medicine, Chiang Mai University, Chiang Mai, Chiang Mai, Thailand

## Abstract

**Background:**

To demonstrate the epidemiological and clinical characteristics of respiratory viral infections among children hospitalized in a tertiary care hospital after COVID-19 pandemic.

**Figure:**
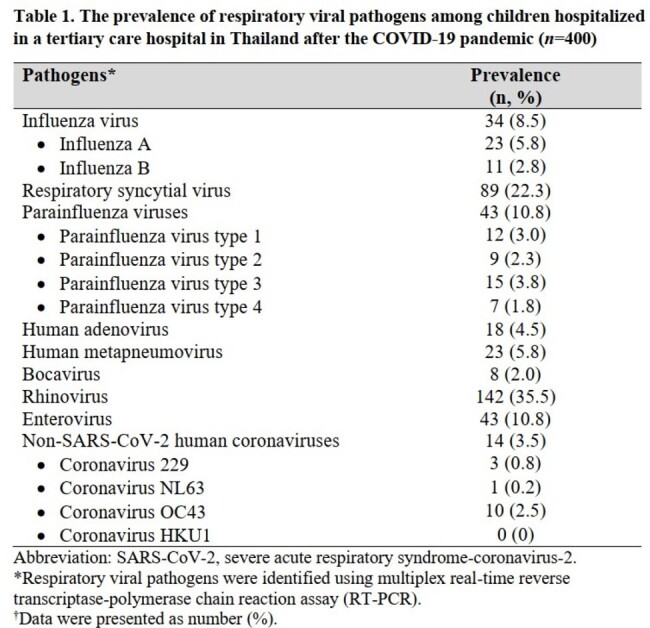

**Methods:**

A cross-sectional study was conducted among children aged 1 month to 15 years who were admitted to the Chiang Mai University Hospital, Thailand due to non-COVID-19 respiratory tract infections (RTIs) after COVID-19 pandemic (Oct 2022 to Sep 2023). Nasopharyngeal specimens were tested for 16 respiratory viruses (RV: influenza A [IAV] and B [IBV] viruses; respiratory syncytial virus [RSV]; parainfluenza virus types 1-4; rhinovirus; enterovirus; human metapneumovirus; human coronaviruses 229, NL63, OC43, HKU1; bocavirus; human adenovirus), using multiplex RT-PCR. The prevalence each RV was demonstrated. Clinical parameters, treatments, and outcomes of children were compared by type of RV. In addition, the prevalence of IAV, IBV and RSV were compared across 3 periods: pre (Jan 2016 to Dec 2019), during (Jan 2020 to Sep 2022), and post COVID-19 pandemic.

**Figure:**
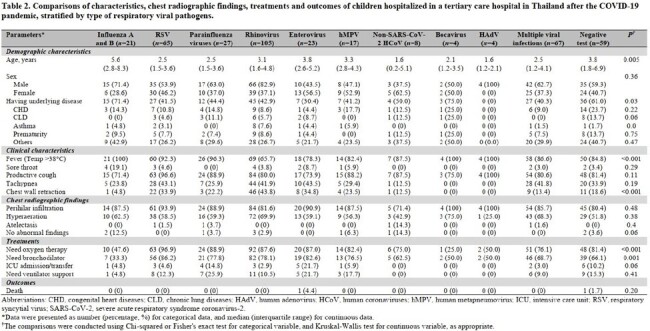

**Results:**

During the study period, 400 children were enrolled; median age 2.9 (IQR 1.5-4.8) years, 59% male, 46% had underlying diseases. On admission, 80% had pneumonia and 5% were admitted to ICUs. Overall, 341 children (85%) were tested positive for ≥ 1 RV (68% single and 17% multiple infections). The prevalence of each RV after the COVID-19 pandemic was shown in Table 1. Compared with other viruses, children with IAV and IVB (*n*=21) had the highest proportion of fever (100%) and sore throat (19%), whereas those with RSV (*n*=65) had the highest proportion of oxygen therapy (97%) and bronchodilator (86%) requirement, and those with enterovirus (*n*=23) had the highest proportion of tachypnea (44%), ICU admission/transfer (22%), ventilator use (22%), and death (4%) (Table 2). Notably, the prevalence of IAV, IBV and RSV significantly declined during the COVID-19 pandemic, and sharply rebounded after the pandemic, particularly for RSV which the prevalence was significantly higher than pre COVID-19 pandemic phase (Figure 1).

**Figure:**
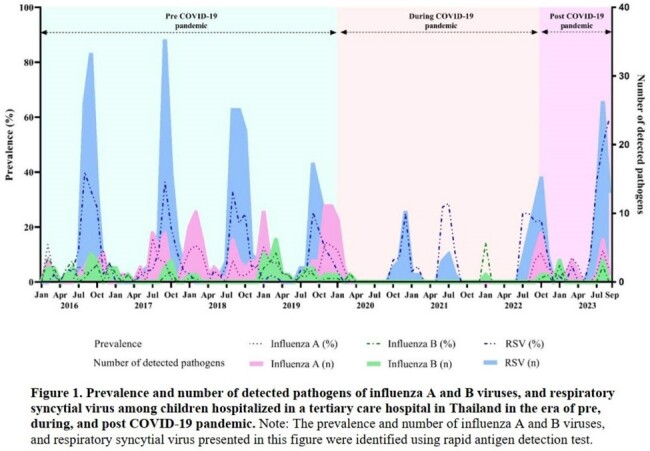

**Conclusion:**

COVID-19 pandemic significantly changed the epidemiology of respiratory viral infections in our setting. Active surveillance is needed to better understand the dynamics and seasonal patterns of RV after the pandemic.

**Disclosures:**

**All Authors**: No reported disclosures

